# Diversity of *Ptychadena* in Rwanda and taxonomic status of *P. chrysogaster* Laurent, 1954 (Amphibia, Anura, Ptychadenidae)

**DOI:** 10.3897/zookeys.356.5878

**Published:** 2013-11-28

**Authors:** J. Maximilian Dehling, Ulrich Sinsch

**Affiliations:** 1 Institut für Integrierte Naturwissenschaften, Abteilung Biologie, Universität Koblenz-Landau, Universitätsstraße 1, D–56070 Koblenz, Germany

**Keywords:** *P. anchietae*, *P. grandisonae*, *P. nilotica*, *P. porosissima*, *P. uzungwensis*, DNA barcoding, systematics

## Abstract

We assess the diversity of *Ptychadena* species in Rwanda based on re-examination of voucher specimens in museum collections and our own data from recent assessment of the species composition of amphibian communities in Rwanda. We recognize five species which we allocate to the following available names: *P. anchietae*, *P. chrysogaster*, *P. nilotica*, *P. porosissima*, and *P. uzungwensis*. We did not find evidence for the presence of *P. grandisonae* and *P. oxyrhynchus* which have been listed for the country. The five species can be distinguished by quantitative morphometrics (discriminant analysis, success rate: 100 %) and a number of qualitative characters of external morphology. We provide an identification key to the Rwandan species and describe the morphology of each species in detail. The taxonomic status and the phylogenetic position of *Ptychadena chrysogaster* are further assessed based on the partial sequence of the mitochondrial 16S rRNA. The species differs genetically from available homologous sequences from congeners by an uncorrected p distance of at least 4.2 % and appears to be most closely related to specimens assigned to *P. porosissima*, *P. mahnerti*, “*P.* aff. *uzungwensis*” and “*P.* aff. *bibroni*”.

## Introduction

Ridged Frogs of the genus *Ptychadena* Boulenger, 1917 are widespread in sub-Saharan Africa where approximately 50 species occur. Species of the genus share a similar general appearance and many are poorly delimited, having been described based on taxonomically doubtful characters. Several species names have been erroneously considered synonyms of others, thus confusing character diagnoses in subsequent accounts; and some taxa were described based on specimens later found to represent more than one species (e.g. Boulenger 1879; Loveridge 1932; Laurent 1954; Guibé and Lamotte 1957; Schmidt and Inger 1959; Lamotte 1967; Poynton 1970; Rödel 2000; Channing 2001; Channing and Howell 2006; Dehling and Sinsch in press). Therefore, even the local/regional diversity of these frogs is often difficult to assess. Herein, we address the diversity of Ridged Frogs in Rwanda. We have recently shown that three species (*Ptychadena anchietae* [Bocage, 1868], *Ptychadena nilotica* [Seetzen, 1855], and *Ptychadena porosissima* [Steindachner, 1867]) inhabit the wetlands along the upper Nile (Dehling and Sinsch in press). Further taxa have been reported from Rwanda and it is currently unclear which species actually occur in the country. Nieden (1913) reported *Ptychadena nilotica* (referred to as *Rana mascareniensis* Duméril & Bibron, 1841) from several localities in Rwanda. Based on his own collections, Laurent (1954) reported *Ptychadena uzungwensis* (Loveridge, 1932) and described *Ptychadena chrysogaster* Laurent, 1954 and *Ptychadena loveridgei* Laurent, 1954 as new species, the latter now being considered a synonym of *Ptychadena porosissima* (Schmidt and Inger 1959). Poynton and Broadley (1985), Channing (2001), and Poynton and Channing (2004) stated that *Ptychadena grandisonae* Laurent, 1954 occurs in Rwanda. Fischer and Hinkel (1992) listed only “*Ptychadena mascareniensis*” [= *Ptychadena nilotica*]. Poynton et al. (2004) stated that *Ptychadena anchietae* was likely to occur in Rwanda but confirmed records were missing. According to Spawls et al. (2006), *Ptychadena chrysogaster*, *Ptychadena mascareniensis*, and *Ptychadena uzungwensis* occur in Rwanda but not *Ptychadena anchietae* and *Ptychadena porosissima*. Branch (2005) included Rwanda in the geographic range of *Ptychadena oxyrhynchus* (Smith, 1849). Recently, we collected *Ptychadena anchietae* in Rwanda and resurrected the name *Ptychadena nilotica* for the populations which occur along the Nile and in Central Kenya and Tanzania and which had been formerly referred to as *Rana mascareniensis* or *Ptychadena mascareniensis* (Sinsch et al. 2012, Dehling and Sinsch in press).

In order to clarify how many and which species occur in Rwanda, we re-examined the specimens of *Ptychadena* in the herpetological collection of the Royal Museum for Central Africa in Tervuren, Belgium (RMCA), on which almost all previous Rwandan records are based. We herein report the results and compare the findings to our own data from recent assessment of the composition of amphibian communities at numerous locations in Rwanda. We further assess the taxonomic status and the phylogenetic position of *Ptychadena chrysogaster* based on examination of most of the available voucher material from Rwanda including the type series and on comparison of the partial sequence of the mitochondrial 16S rRNA gene with homologous sequences of its congeners.

## Material and methods

### Morphological examination

We examined voucher specimens deposited at RMCA. Additional specimens including our recently collected material are deposited in the collection of the Zoologisches Forschungsmuseum Alexander Koenig, Bonn, Germany (ZFMK). See Appendix 1 for a complete list of examined specimens.

For the morphological analysis, we took the following 18 measurements to the nearest 0.1 mm using digital calipers, following Dehling and Sinsch (in press): (1) Snout-vent length (SVL); (2) tibiofibula length (TFL, measured with both knee and tibio-tarsal articulation flexed); (3) thigh length (THL, from vent to knee with thigh being held vertically to median body plane and knee flexed); (4) total hindlimb length (LEG, from vent to tip of fourth toe with leg fully extended and being held vertically to median body plane); (5) tarsus + foot length (TarL, from tibio-tarsal articulation to tip of fourth toe); (6) foot length (FOT, from proximal end of inner metatarsal tubercle to tip of fourth toe); (7) forearm + hand length (ARM, distance from elbow to tip of third finger); (8) hand length (HND, distance from proximal end of inner palmar tubercle to tip of third finger); (9) head width (HW, measured at the level of the jaw joint); (10) head length (HL, distance from posterior end of mandible to tip of snout); (11) interorbital distance (IO, shortest distance between upper eyelids); (12) upper eyelid width (EW); (13) horizontal eye diameter (ED); (14) horizontal tympanum diameter (TD); (15) eye to nostril distance (EN, distance between anterior margin of eye and centre of nostril); (16) nostril to snout distance (NS, distance between centre of nostril and tip of snout); (17) snout length (SL, distance between anterior margin of eye to tip of snout); (18) internarial distance (NN, distance between centres of nostrils). To avoid an inter-observer bias, all measurements were taken by JMD. Additionally, we recorded the following qualitative characters: (1) position of external vocal sac aperture in males; (2) number of longitudinal dorsal dermal ridges; (3) texture of ventral skin; (4) extent of nuptial pads in males; (5) number of supernumerary metacarpal tubercles; (6) size and shape of thenar and palmar tubercles; (7) extent of toe webbing; (8) presence of outer metatarsal tubercle; (9) relative size of inner metatarsal tubercle; (10) ventral colouration; (11) presence of light line on dorsal face of tibia; (12) presence of light band on dorsum; (13) presence of dark brown stripe on preaxial side of tarsus; (14) colour of external dorsal fold; (15) colour pattern on postaxial side of femur. Sex of males was determined by presence of secondary sexual characteristics (vocal slits, nuptial pads), that of females by either examination of gonads through dissection or size (female if larger than smallest 10 percent of adult males). The webbing formulae are given as proposed by Myers and Duellman (1982). Terminology for dermal dorsal ridges and orientation of external vocal sac aperture follows Perret (1979).

### Statistical Analyses

Descriptive statistics depended on the outcome of the test for normality. Normally distributed data were described by the arithmetic mean and corresponding standard error and/or range, those deviating significantly by median and range. Principal component analyses were run on the morphometric data set including 18 variables and 89 observations each (*Ptychadena anchietae*: 15 males, 3 females; *Ptychadena chrysogaster*: 13 males, 10 females; *Ptychadena nilotica*: 13 males, 10 females; *Ptychadena porosissima*: 11 males, 7 females; *Ptychadena uzungwensis*: 6 males, 1 female). We compared the scores obtained for the principal components 2 and 3 describing shape to distinguish taxa without *a priori* assignment to taxa. The morphometric distances were adjusted for SVL by calculating a linear regression of each variable against SVL and storing the residuals as representatives of size-independent shape variables. This transformed data set was used for discriminant analyses with taxa as predefined groups to optimize distinction. To account for sexual dimorphism, discriminant analyses were run separately for males (n=58) and females (n=31). Significance level was set at alpha = 0.05. All calculations were based on the procedures of the program package STATGRAPHICS centurion for Windows, version XV.

### DNA barcoding and phylogenetic analyses

We isolated DNA from a liver tissue sample from a specimen of *Ptychadena chrysogaster* (ZFMK 58797), collected in southern Rwanda by H. Hinkel in 1993. DNA was used to sequence a fragment of the 16S mitochondrial rRNA gene, a universal marker to barcode amphibian species (Vences et al. 2005). Protocols of DNA extraction, PCR, purification, and sequencing follow Dehling and Sinsch (in press). The obtained sequence was compared with those in GenBank using a standard nucleotide-nucleotide BLAST search and with our own sequences from Rwandan specimens and was incorporated into an existing alignment (see Dehling and Sinsch in press for a list of sequences and GenBank Accession numbers). Editing and alignment were completed in MEGA5 (Tamura et al. 2011). Sequences were trimmed to the same length. The final alignment consisted of 548 base pairs. Calculations of pairwise distances and phylogenetic analysis (Maximum Likelihood) were carried out in MEGA5. Maximum Likelihood analysis was run using the GTR + G + I model and the Nearest-Neighbor-Interchange with 1000 bootstrap replicates.

## Results

**Figure 1.**
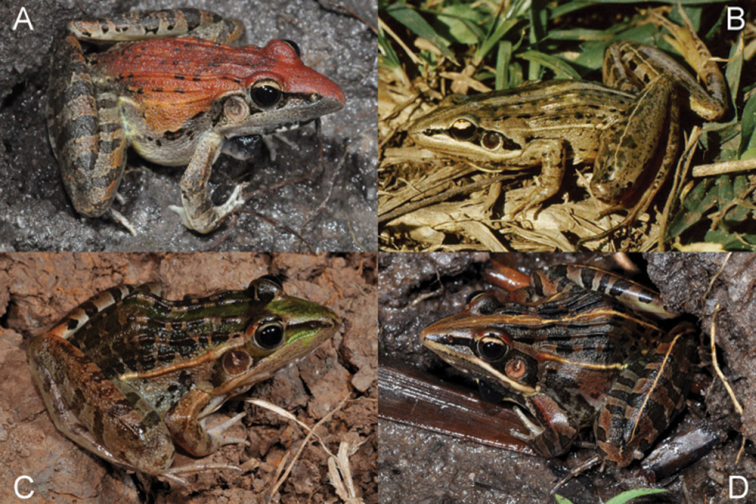
Males of *Ptychadena* from Rwanda in life. **A** *Ptychadena anchietae* **B** *Ptychadena chrysogaster* [Foto: E. Fischer] **C** *Ptychadena nilotica* **D** *Ptychadena porosissima*.

**Figure 2.**
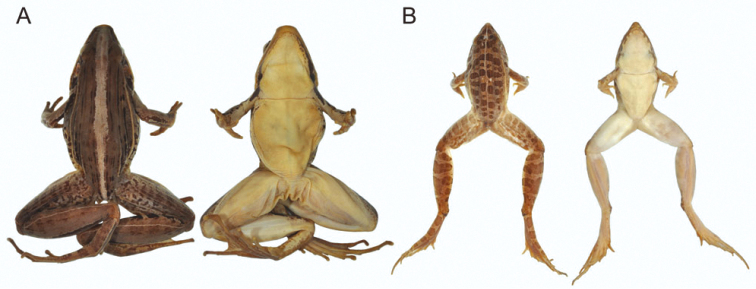
**A** Preserved female holotype of *Ptychadena chrysogaster* (RMCA 109096) from Lac Karago, Rwanda; dorsal view (left) and ventral view (right) **B** Preserved male specimen of *Ptychadena uzungwensis* (RMCA 108993-108997) from Munini, Rwanda; dorsal view (left) and ventral view (right). Not to scale.

Examination of specimens suggested that five morphologically distinct species were present in Rwanda to which we assign the following names: *Ptychadena anchietae*, *Ptychadena chrysogaster*, *Ptychadena nilotica*, *Ptychadena porosissima*, and *Ptychadena uzungwensis* (Figures 1 and 2). For allocation of specimens to *Ptychadena anchietae*, *Ptychadena nilotica*, and *Ptychadena porosissima* and discussion thereof see Dehling and Sinsch (in press). The examined material included type specimens of both of *Ptychadena chrysogaster* and *Ptychadena uzungwensis* (Appendix 1). Allocation of other specimens to the latter two species is based on direct comparison with the type material. We re-assigned several specimens that had been deposited in the museum collections under wrong names. Noteworthy are two of the paratypes of *Ptychadena chrysogaster* (RMCA 41989, 41994) which belong in fact to *Ptychadena porosissima*.

### Morphological differentiation

**Figure 3.**
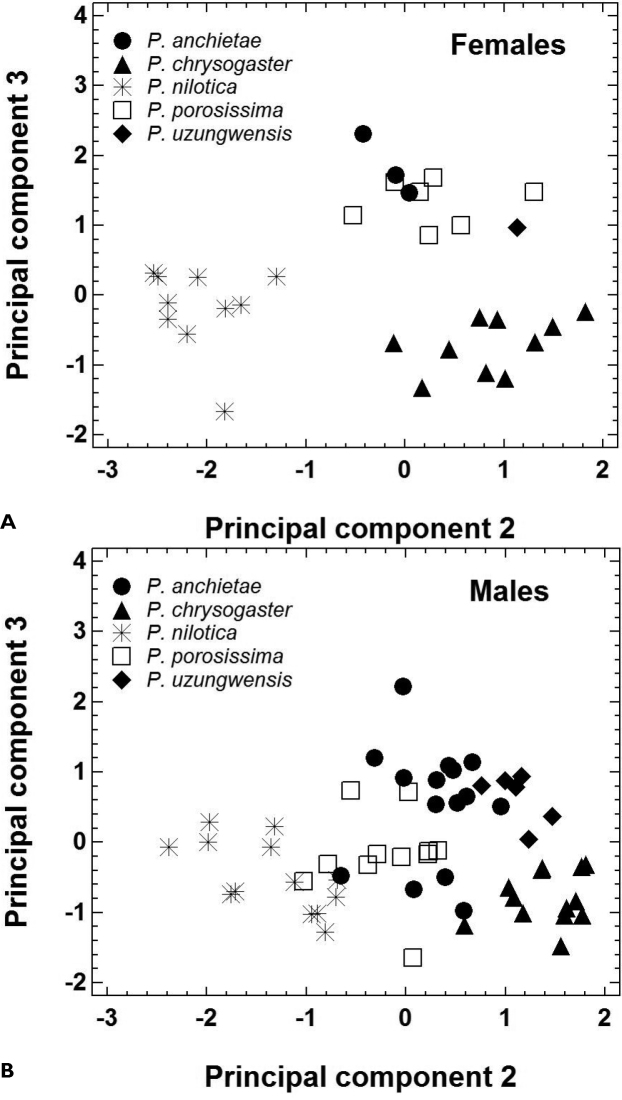
Morphological shape differentiation among 89 specimens representing five *Ptychadena* species, as assessed by principal component analysis (Table 3). **A** Individual scores obtained for 31 females **B** Individual scores obtained for 58 males.

**Figure 4.**
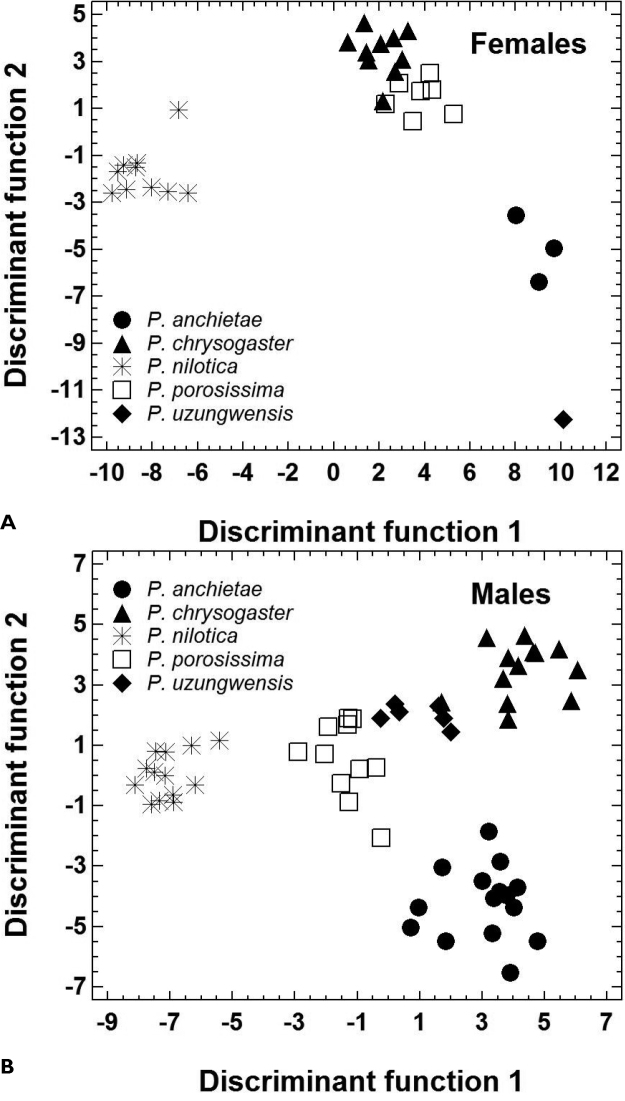
Morphological shape differentiation among 89 specimens representing five *Ptychadena* species, as assessed by discriminant analyses (Statistical details are given in Table 4). **A** Individual scores obtained for 31 females **B** Individual scores obtained for 58 males.

The morphometric features of the five species are summarized in Table 2. Principal component analysis yielded three PCs accounting for 89.6% of total variation (Table 3A). PC1 represented variation in size, whereas the shape-related PC2 and PC3 were mainly loaded by features describing head morphology (Table 3B). In females, PC 2 unequivocally distinguished *Ptychadena nilotica* from the other taxa, and PC 3 unequivocally distinguished *Ptychadena chrysogaster* from *Ptychadena anchietae*, *Ptychadena porosissima*, and *Ptychadena uzungwensis* (Figure 3A). Also, *Ptychadena anchietae* and *Ptychadena uzungwensis* could be distinguished from each other. However, both species were represented by only few individuals (three and one, respectively) in the analysis. The two species did not differ significantly in shape from *Ptychadena porosissima* (Figure 3A). A similar pattern was observed in the analysis of the males (Figure 3B) but males of all five species were generally more similar to each other in shape. Males of *Ptychadena nilotica* could be distinguished unequivocally from males of *Ptychadena chrysogaster* and *Ptychadena uzungwensis* but not from some of the males of *Ptychadena anchietae* and *Ptychadena porosissima* (Figure 3B). Males of *Ptychadena chrysogaster* could be distinguished from males of all other species but some of the males of *Ptychadena anchietae* and *Ptychadena uzungwensis* were very similar in shape (Figure 3B). Males of *Ptychadena anchietae* did not differ significantly in shape from males of *Ptychadena nilotica*, *Ptychadena porosissima*, and *Ptychadena uzungwensis*. Gender-specific discriminant analyses based on the residuals of 17 SVL-adjusted morphometric variables had a classification success of 100 % among the five species in both males and females (Table 4A, B, C, Figure 4A, B).

**Figure 5.**
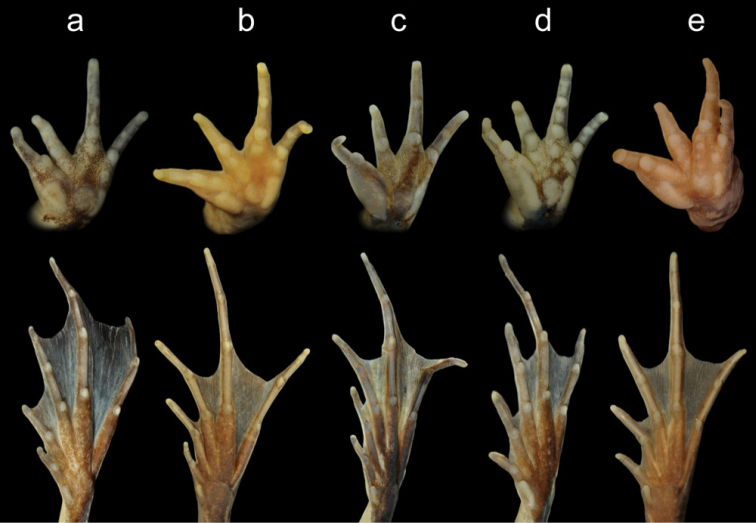
Volar view of hands (top) and plantar view of feet (bottom) of males of *Ptychadena anchietae* (**a**), *Ptychadena chrysogaster* (**b**), *Ptychadena nilotica* (**c**), *Ptychadena porosissima* (**d**), and *Ptychadena uzungwensis* from Rwanda. See also Table 1.

The five Rwandan species can be distinguished unequivocally from each other using a combination of qualitative morphological characters (Table 1, Figure 5; see also Dehling and Sinsch in press). An identification key based on these characters is given below. Detailed morphological descriptions of the species are in Appendix 2.

**Table 1.** Distinguishing qualitative characters of *Ptychadena* species from Rwanda.

Species	*Ptychadena anchietae*	*Ptychadena chrysogaster*	*Ptychadena nilotica*	*Ptychadena porosissima*	*Ptychadena uzungwensis*
relative length of Toes III and V	tips reaching to knee or slightly beyond, distal subarticular tubercle never reaching knee	tips reaching beyond knee, distal subarticular tubercle reaching knee	tips reaching beyond knee, distal subarticular tubercle reaching knee or beyond	tips reaching to knee or slightly beyond, distal subarticular tubercle never reaching knee	tips reaching to knee or slightly beyond, distal subarticular tubercle never reaching knee
position of vocal sac aperture	inferior, at ventral edge of arm insertion	inferior, at ventral edge of arm insertion	superior, above dorsal edge of arm insertion	inferior, at ventral edge of arm insertion	semi-inferior, at level of centre of arm insertion
spiny tubercles on venter	absent	present in males, very small	absent	present in males, comparatively large	present in males, very small
median dorsal ridge on snout	absent	absent	absent	absent	present
outer metatarsal tubercle	very faintly visible	very faintly visible, rarely distinct	distinctly present, rarely faintly visible	faintly visible, rarely distinct	faintly visible
inner metatarsal tubercle size (Fig. 5)	about half the length of metatarsus of Toe I	less than half the length of metatarsus of Toe I	less than half the length of metatarsus of Toe I	more than half the length of metatarsus of Toe I	about half the length of metatarsus of Toe I
supernumerary metacarpal tubercles (Fig. 5)	one below each finger	one below each finger	only one below Finger IV, often indistinct	one below Fingers I, II, and IV; two, rarely one below Finger III	one below Fingers I and IV, two below Finger II, two to four below Finger III
palmar and thenar tubercles (Fig. 5)	inner and outer palmar tubercle more or less equal in length; thenar tubercle oval, slightly longer than palmar tubercles	outer palmar tubercle longer than inner; thenar tubercle elongate, about as long as outer palmar tubercle	outer palmar tubercle longer than inner; thenar tubercle elongate, about as long as outer palmar tubercle	outer palmar tubercle longer than inner; thenar tubercle elongate, longer than outer palmar tubercle	inner and outer palmar tubercle more or less equal in length; thenar tubercle elongate, longer than palmar tubercles
toe webbing (Fig. 5)	I0.5-2II0.5-2III(0.5-1)-2IV2-0.5V	I2-2.5II(1.5-1.75)-3III(2-2-)(3.25-3+)IV3-(1.5-2)V	I(1.5-1.75)-(2-2.25)II1.5-(2.75-3)III(1.75-2)-3IV2.75-(1-1.5)V	I(1.75-2)-2.25II1.5-3III1.75-(3-3.25)IV3-(1-1.5)V	I2-(2.25-2.5)II1.5-3^-^III(1.75-2^-^)-3IV3-(1^+^-1.25)V
ventral colouration	head white, trunk yellow	head and trunk yellow	head white, mottled with grey; trunk yellow	head and trunk yellow	colours in life unreported
dark brown stripe on preaxial side of tibia	absent	present, continuous or almost continuous	absent in most specimens; few specimens with dark mottling, not forming continuous stripe	absent	absent
light tibial line (Figs 1 & 2)	absent	usually present, rarely absent	present or absent	present	absent
light dorsal band	absent	usually present, rarely absent	present or absent	present or absent	present
dark spots on dorsum<br/> (Figs 1 & 2)	usually absent; if present, small and narrow	usually present, small and narrow, sometimes forming longitudinal lines; rarely absent	present, large and wide, sometimes fused with neighboring ones	present, large and wide, sometimes fused with neighboring ones	present, large and wide, often fused with neighboring ones
light, prominent dorsolateral fold (Figs 1 & 2)	usually absent	present	present	present	present
Colour pattern on postaxial side of femur	irregularly delimited, reticulated, longitudinal bands, alternately yellow and dark brown coloured	irregularly delimited, reticulated, longitudinal dark bands on light background; colours in life unreported	relatively sharply delimited longitudinal bands, alternately yellow and black coloured	yellow spots diffusely arranged in longitudinal rows on dark brown background	irregularly delimited, reticulated, longitudinal light bands on dark background; colours in life unreported

**Table 2.** Morphometric features of *Ptychadena* species from Rwanda. Data are given as arithmetic means and minimum and maximum values (in mm).

Morphometric character	*Ptychadena anchietae*		*Ptychadena chrysogaster*		*Ptychadena nilotica*		*Ptychadena porosissima*		*Ptychadena uzungwensis*	
	Males<br/> N = 15	Females<br/> N = 3	Males<br/> N = 75/14*	Females<br/> N = 23/11*	Males<br/> N = 13	Females<br/> N = 10	Males<br/> N = 11/20*	Females<br/> N = 9	Males<br/> N = 6	Females<br/> N = 1
Snout-vent length	40.4<br/> (38.0–42.4)	49.0<br/> (46.7–51.3)	43.3<br/> (36.3–49.5)	53.7<br/> (48.0–57.7)	42.0<br/> (37.2–45.2)	49.1<br/> (45.6–53.1)	41.2*<br/> (37.3–44.5)	46.4<br/> (39.0–52.1)	34.7<br/> (33.3–35.7)	43.3<br/> -
Hindlimb length	79.6<br/> (74.2–84.9)	98.9<br/> (96.0–101.2)	88.1<br/> (83.5–93.0)	108.2<br/> (102.3–114.1)	77.2<br/> (70.1–85.2)	89.6<br/> (78.0–103.9)	78.2*<br/> (72.8–85.5)	88.3<br/> (74.3–94.1)	66.7<br/> (61.9–72.7)	81.2<br/> -
Femur length	23.0<br/> (21.9–24.5)	29.0<br/> (28.3–29.6)	23.9<br/> (22.5–25.0)	29.9<br/> (28.1–32.0)	21.5<br/> (19.4–24.0)	25.5<br/> (23.0–28.5)	21.7*<br/> (20.2–24.1)	24.4<br/> (19.8–27.6)	18.7<br/> (17.2–19.6)	23.1<br/> -
Tibiofibula length	26.3<br/> (24.4–28.0)	33.1<br/> (31.9–33.8)	28.4<br/> (24.6–32.0)	35.2<br/> (32.4–38.5)	23.4<br/> (21.1–26.1)	27.5<br/> (23.6–32.1)	24.7*<br/> (23.3–26.5)	28.7<br/> (25.2–31.0)	21.7<br/> (20.3–23.4)	26.9<br/> -
Tarsus length	34.5<br/> (31.6–36.5)	42.7<br/> (40.6–44.1)	40.9*<br/> (38.9–44.1)	49.3*<br/> (46.5–51.3)	36.2<br/> (32.4–40.1)	42.8<br/> (35.9–49.6)	35.5<br/> (33.3–38.8)	40.0<br/> (32.6–43.5)	30.5<br/> (27.9–32.2)	36.0<br/> -
Foot length	24.7<br/> (22.4–26.1)	30.5<br/> (29.1–31.3)	28.4<br/> (24.2–30.4)	34.5<br/> (32.9–36.1)	25.7<br/> (22.8–28.2)	29.3<br/> (25.6–33.9)	24.4<br/> (23.0–26.7)	27.4<br/> (23.1–29.3)	21.0<br/> (19.5–21.9)	28.6<br/> -
Forelimb length	16.9<br/> (15.9–18.1)	21.0<br/> (20.0–21.7)	18.3*<br/> (17.4–19.1)	22.0*<br/> (20.9–23.9)	17.7<br/> (16.0–19.3)	20.5<br/> (17.9–23.7)	17.2<br/> (16.0–18.6)	18.7<br/> (16.1–20.9)	13.5<br/> (12.8–14.4)	16.3<br/> -
Hand length	10.0<br/> (9.5–10.7)	12.3<br/> (12.1–12.6)	10.4*<br/> (9.6–11.1)	12.4*<br/> (11.8–13.3)	10.4<br/> (9.5–11.6)	11.9<br/> (10.6–13.8)	9.7<br/> (8.9–10.8)	10.7<br/> (9.1–11.7)	7.9<br/> (7.4–8.5)	9.0<br/> -
Head width	13.8<br/> (12.5–15.4)	16.7<br/> (16.4–17.0)	13.9<br/> (12.8–14.7)	16.9<br/> (15.4–17.5)	13.8<br/> (12.4–15.9)	16.4<br/> (14.2–18.8)	14.1<br/> (13.1–15.0)	15.1<br/> (12.5–17.1)	11.4<br/> (11.0–11.7)	13.8<br/> -
Head length	15.5<br/> (14.1–17.6)	18.7<br/> (18.2–19.1)	15.6*<br/> (14.7–16.4)	18.6*<br/> (17.9–19.8)	16.2<br/> (14.7–18.4)	18.3<br/> (16.6–20.4)	15.5<br/> (14.2–17.8)	17.1<br/> (13.6–19.1)	13.3<br/> (12.3–14.0)	16.1<br/> -
Interorbital distance	2.8<br/> (2.4–3.1)	3.1<br/> (3.0–3.2)	3.5*<br/> (3.1–3.9)	4.1*<br/> (3.8–4.5)	2.2<br/> (1.9–2.5)	2.5<br/> (2.2 - 2.7)	2.6<br/> (2.3–2.7)	3.1<br/> (2.8–3.6)	2.7<br/> (2.4–3.1)	2.9<br/> -
Eyelid width	2.8<br/> (2.5–3.1)	3.2<br/> (3.0–3.3)	2.7*<br/> (2.4–3.0)	3.3*<br/> (2.9–3.7)	2.7<br/> (2.3–3.2)	3.0<br/> (2.4–3.5)	2.8<br/> (2.3–3.1)	3.1<br/> (2.6–3.5)	2.6<br/> (2.3–2.8)	2.4<br/> -
Eye diameter	4.3<br/> (3.6–4.9)	5.2<br/> (5.0–5.5)	4.3<br/> (3.7–4.9)	5.0<br/> (4.6–5.6)	4.5<br/> (4.1–5.1)	5.1<br/> (4.8–5.5)	4.2<br/> (3.9–4.5)	4.6<br/> (3.9–5.1)	3.9<br/> (3.6–4.1)	4.5<br/> -
Tympanum diameter	3.3<br/> (2.9–3.6)	4.1<br/> (3.7–4.6)	3.7<br/> (3.2–4.2)	4.5<br/> (4.3–5.1)	3.7<br/> (3.3–4.1)	4.1<br/> (3.7–4.7)	3.1<br/> (2.9–3.49	3.6<br/> (3.3–3.9)	2.9<br/> (2.5–3.3)	3.5<br/> -
Eye–nostril distance	4.1<br/> (3.7–4.3)	5.2<br/> (4.9–5.7)	3.9*<br/> (3.5–4.4)	4.6*<br/> (4.0–5.0)	3.6<br/> (3.4–4.0)	4.2<br/> (3.7–4.9)	3.5<br/> (3.1–3.9)	4.2<br/> (3.6–4.7)	3.5<br/> (3.2–3.6)	4.2<br/> -
Snout–nostril distance	3.5<br/> (2.9–4.0)	4.4<br/> (4.3–4.5)	3.9*<br/> (3.5–4.3)	4.3*<br/> (3.9–4.9)	3.4<br/> (2.9–3.8)	3.7<br/> (3.3–4.1)	3.4<br/> (2.9–3.7)	4.1<br/> (2.8–4.7)	3.6<br/> (3.2–3.9)	4.3<br/> -
Internarial distance	3.9<br/> (3.5–4.3)	4.8<br/> (4.7–4.9)	4.3*<br/> (4.0–4.5)	4.9*<br/> (4.5–5.2)	3.4<br/> (3.0–3.6)	3.7<br/> (2.0–4.5)	3.6<br/> (3.4–4.0)	4.3<br/> (3.7–4.7)	3.0<br/> (2.9–3.2)	3.7<br/> -
Snout length	7.5<br/> (6.6–8.1)	9.5<br/> (9.0–10.1)	7.4*<br/> (7.0–7.9)	8.7*<br/> (8.4–9.4)	7.0<br/> (6.3–7.7)	7.9<br/> (7.1–9.3)	7.0<br/> (6.3–7.8)	8.1<br/> (6.2–10.1)	6.7<br/> (6.4–7.0)	8.0<br/> -

**Table 3.** Principal component Analysis based on 18 standardized morphometric features of 89 specimens belonging to five *Ptychadena* species from Rwanda. Morphometric parameters accounting strongly for discrimination among species are highlighted in **bold**.

**A: Statistical significance**			
**Principal component**	**Eigen-value**	**Relative percentage**	**Cumulative percentage**
1	13.84	76.9	76.9
2	1.46	8.1	85.0
3	0.82	4.6	89.6
**B: Standardized coefficients of the principal components**			
**Parameter**	**Principal component 1**	**Principal component 2**	**Principal component 3**
Snout-vent length	0.256	-0.107	-0.084
tibiofibula length	0.253	0.212	-0.011
foot length	0.255	0.046	-0.278
tarsus + foot length	0.257	0.056	-0.246
total hindlimb length	0.261	0.109	-0.137
thigh length	0.260	0.048	-0.033
forearm + hand length	0.253	-0.164	-0.210
hand length	0.244	-0.231	-0.139
head width	0.243	-0.224	-0.016
head length	0.232	**-0.319**	0.105
interorbital distance	0.171	**0.578**	-0.237
upper eyelid width	0.201	-0.142	0.272
horizontal eye diameter	0.211	-0.325	0.162
horizontal tympanum diameter	0.231	-0.112	-0.284
eye to nostril distance	0.228	0.016	**0.353**
nostril to snout distance	0.195	**0.326**	**0.412**
snout length	0.234	0.070	**0.469**
internarial distance	0.227	**0.324**	0.072

**Table 4A.** Gender-specific discriminant functions based on 17 SVL-adjusted morphometric features (residuals) to distinguish among five *Ptychadena* species from Rwanda. Statistical significance:

Discriminant function	Eigen-value	Relative percentage	Canonical correlation	Wilks Lambda	Chi-squared	Degrees of freedom	Statistical significance
Male 1	19.79	58.76	0.975	0.0003	362.2	68	P < 0.0001
Male 2	8.49	25.23	0.945	0.0079	222.6	48	P < 0.0001
Male 3	3.29	9.77	0.875	0.0750	119.0	30	P < 0.0001
Male 4	2.10	6.24	0.823	0.3223	52.0	14	P < 0.0001
Female 1	45.49	65.38	0.989	0.00005	187.7	68	P < 0.0001
Female 2	14.84	21.33	0.967	0.0023	114.7	48	P < 0.0001
Female 3	6.87	9.89	0.934	0.0377	62.2	30	P < 0.0001
Female 4	2.36	3.40	0.838	0.2973	23.0	14	P = 0.0596

**Table 4B.** Gender-specific discriminant functions based on 17 SVL-adjusted morphometric features (residuals) to distinguish among five *Ptychadena* species from Rwanda. Morphometric parameters accounting strongly to discrimination among species are highlighted in **bold**. Standardized coefficients of the discriminant functions:

parameter (residuals)	discriminant function 1<br/> (males)	discriminant function 2<br/> (males)	discriminant function 3<br/> (males)	discriminant function 4<br/> (males)	discriminant function 1<br/> (females)	discriminant function 2<br/> (females)	discriminant function 3<br/> (females)	discriminant function 4<br/> (females)
tibiofibula length	**0.909**	-0.221	0.302	0.871	0.824	-0.143	0.196	0.384
foot length	0.268	-0.253	0.292	**-1.332**	**2.079**	**-3.834**	**-2.308**	**-0.942**
tarsus + foot length	**-0.798**	**2.031**	-0.026	**1.308**	**-3.311**	**4.158**	**1.119**	-0.410
total hindlimb length	0.310	-0.029	-0.147	**-0.929**	-0.617	0.188	0.361	**0.999**
thigh length	0.161	**-1.001**	0.367	-0.108	0.750	0.009	-0.268	0.196
forearm + hand length	-0.172	0.142	**-1.406**	-0.005	0.468	-0.492	0.483	0.105
hand length	**-0.669**	-0.506	0.383	-0.189	-0.879	-0.007	0.088	0.328
head width	0.009	-0.137	-0.370	0.326	-0.778	**0.997**	0.472	0.534
head length	-0.282	-0.341	0.252	-0.389	-0.293	**-1.250**	-0.563	-0.734
interorbital distance	0.366	0.144	0.285	0.136	**1.030**	0.561	-0.269	-0.019
upper eyelid width	-0.022	0.315	-0.221	0.328	**1.579**	0.473	**0.810**	-0.234
horizontal eye diameter	-0.296	-0.048	0.420	-0.193	**1.013**	0.232	**0.808**	0.089
eye to nostril distance	0.417	-0.532	0.473	-0.287	**1.016**	-0.555	0.242	-0.166
nostril to snout distance	0.072	0.529	0.225	0.124	1.162	-0.599	0.727	-0.818
snout length	-0.291	-0.063	-0.123	0.422	-0.176	0.035	-0.337	0.767
internarial distance	**0.666**	0.135	**-0.861**	-0.567	0.594	0.087	-0.026	-0.213
horizontal tympanum diameter	-0.052	-0.157	**0.707**	-0.187	**-1.365**	0.088	-0.621	0.488
Constant	0.909	-0.222	0.302	0.871	0.824	-0.143	0.196	0.384

**Table 4C.** Gender-specific discriminant functions based on 17 SVL-adjusted morphometric features (residuals) to distinguish among five *Ptychadena* species from Rwanda. Classification success.

predicted species<br/> actual species	*Ptychadena anchietae*	*Ptychadena chrysogaster*	*Ptychadena nilotica*	*Ptychadena porosissima*	*Ptychadena uzungwensis*
*Ptychadena anchietae*. male<br/> female	15 (100%)<br/> 3 (100%)	0	0	0	0
*Ptychadena chrysogaster*. male<br/> female	0	13 (100%)<br/> 10 (100%)	0	0	0
*Ptychadena nilotica*. male<br/> female	0	0	13 (100%)<br/> 10 (100%)	0	0
*Ptychadena porosissima*. male<br/> female	0	0	0	11 (100%)<br/> 7 (100%)	0
*Ptychadena uzungwensis*. male<br/> female	0	0	0	0	6 (100%)<br/> 1 (100%)

### Key to the Rwandan species of Ptychadena

**Table:** 

1	external vocal sac apertures and nuptial pads on dorsal side of metacarpals and phalanges of Fingers I–III present	adult males...2
–	external vocal sac apertures and nuptial pads absent	adult females and subadults...6
2	vocal sac aperture superior; only one supernumerary metacarpal tubercle proximal to Finger IV, often indistinct; longitudinal, alternately black and yellow coloured bands on postaxial side of femur (spiny tubercles on venter absent; inner metatarsal tubercle less than half the length of metatarsus of Toe I; distal subarticular tubercles of Toes III and V reaching to knee; toe webbing I(1.5–1.75)-(2–2.25)II1.5-(2.75–3)III(1.75–2)-3IV2.75-(1–1.5)V; ventral side of head white, mottled with grey)	*Ptychadena nilotica*
–	vocal sac aperture inferior or semi-inferior; at least one supernumerary metacarpal tubercle proximal to each finger; colouration on postaxial side of femur different	3
3	spiny tubercles on venter absent; toe webbing reaching distal phalanx on postaxial sides of Toes I, II, and III and on preaxial side of Toe V; external dorsal ridge usually not light and prominent (vocal sac aperture inferior; distal subarticular tubercles of Toes III and V never reaching knee; inner metatarsal tubercle about half the length of metatarsus of Toe I; ventral side of head white, trunk yellow; light tibial line and light dorsal band absent; dark spots on dorsum usually absent, if present, small and narrow; irregularly delimited, reticulated, longitudinal, alternately yellow and dark brown coloured bands on postaxial side of femur)	*Ptychadena anchietae*
–	spiny tubercles on venter present; toe webbing not reaching distal phalanges on toes; external dorsal ridge light and prominent	4
4	median dorsal ridge extending to level between nostrils on dorsal side of snout; vocal sac aperture semi-inferior; two supernumerary metacarpal tubercles proximal to Finger II; inner and outer palmar tubercle more or less equal in length; inner metatarsal tubercle about half the length of metatarsus of Toe I	*Ptychadena uzungwensis*
–	median dorsal ridge extending to level between eyelids only; vocal sac aperture inferior; one supernumerary metacarpal tubercle proximal to Finger II; outer palmar tubercle longer than inner; inner metatarsal tubercle either more than or less than half the length of metatarsus of Toe I	5
5	foot large, tips of Toes III and V reaching distinctly beyond knee, their distal subarticular tubercles reaching knee; ventral tubercles tiny, hardly visible with naked eye; inner metatarsal tubercle less than half the length of metatarsus of Toe I; dark brown stripe present on preaxial side of tibia; thenar tubercle approximately as long as outer palmar tubercle; webbing not reaching beyond distal subarticular tubercle on postaxial side of Toe III; dorsal spots small and narrow; irregularly delimited, reticulated, longitudinal dark bands on light background on postaxial side of femur	*Ptychadena chrysogaster*
–	foot smaller, tips of Toes III and V at most reaching slightly beyond knee, their distal subarticular tubercles not reaching knee; ventral tubercles large, visible with naked eye, palpable with finger; inner metatarsal tubercle more than half the length of metatarsus of Toe I; dark brown stripe absent on preaxial side of tibia; thenar tubercle longer than outer palmar tubercle; webbing reaching beyond distal subarticular tubercle on postaxial side of Toe III; dorsal spots large and wide; yellow spots, diffusely arranged in longitudinal rows on dark brown background on postaxial side of femur	*Ptychadena porosissima*
6	median dorsal ridge extending to level between nostrils on dorsal side of snout; two supernumerary metacarpal tubercles proximal to Finger II (inner metatarsal tubercle about half the length of metatarsus of Toe I; distal subarticular tubercles of Toes III and V not reaching to knee; toe webbing I2-(2.25–2.5)II1.5-3^-^III(1.75–2)-3IV3-(1^+^–1.25)V; light tibial line absent; light dorsal band present; dark spots on dorsum large and wide, often fused with neighboring ones; light, prominent dorsolateral fold present)	*Ptychadena uzungwensis*
–	median dorsal ridge extending to level between eyelids only; one or no supernumerary metacarpal tubercle proximal to Finger II	7
7	toe webbing reaching to distal phalanx on postaxial sides of Toes I, II, and III and on preaxial side of Toe V; light prominent external dorsal ridge usually absent; inner metatarsal tubercle about half the length of metatarsus of Toe I (tips of Toes III and V at most reaching slightly beyond knee, their distal subarticular tubercles not reaching knee; ventral side of head white, trunk yellow; light tibial line and light dorsal band absent; dark spots on dorsum usually absent, if present, small and narrow; irregularly delimited, reticulated, longitudinal, alternately yellow and dark brown coloured bands on postaxial side of femur)	*Ptychadena anchietae*
–	toe webbing not reaching to distal phalanx on toes; light prominent external dorsal ridge present; inner metatarsal tubercle either less than or more than half the length of metatarsus of Toe I	8
8	inner metatarsal tubercle more than half the length of metatarsus of Toe I; tips of Toes III and V at most reaching slightly beyond knee, their distal subarticular tubercles not reaching knee; thenar tubercle longer than outer palmar tubercle; yellow spots, diffusely arranged in longitudinal rows on dark brown background on postaxial side of femur	*Ptychadena porosissima*
–	inner metatarsal tubercle less than half the length of metatarsus of Toe I; tips of Toes III and V reaching distinctly beyond knee, their distal subarticular tubercles reaching knee; thenar tubercle about as long as outer palmar tubercle; colouration on postaxial side of femur not consisting of spots	9
9	dorsal spots small and narrow; one supernumerary metacarpal tubercle proximal to each finger; ventral side of head and chest yellow; dark brown stripe present on preaxial side of tibia; irregularly delimited, reticulated, longitudinal dark bands on light background on postaxial side of femur; webbing not reaching beyond subarticular tubercle on Toe I	*Ptychadena chrysogaster*
–	dorsal spots large and wide; only one supernumerary metacarpal tubercle proximal to Finger IV, often indistinct; ventral side of head and chest white; dark brown stripe on preaxial side of tibia absent, few specimens with dark mottling, not forming continuous stripe; longitudinal, alternately black and yellow coloured bands on postaxial side of femur; webbing reaching beyond subarticular tubercle on Toe I	*Ptychadena nilotica*

### Phylogenetic analyses

**Figure 6.**
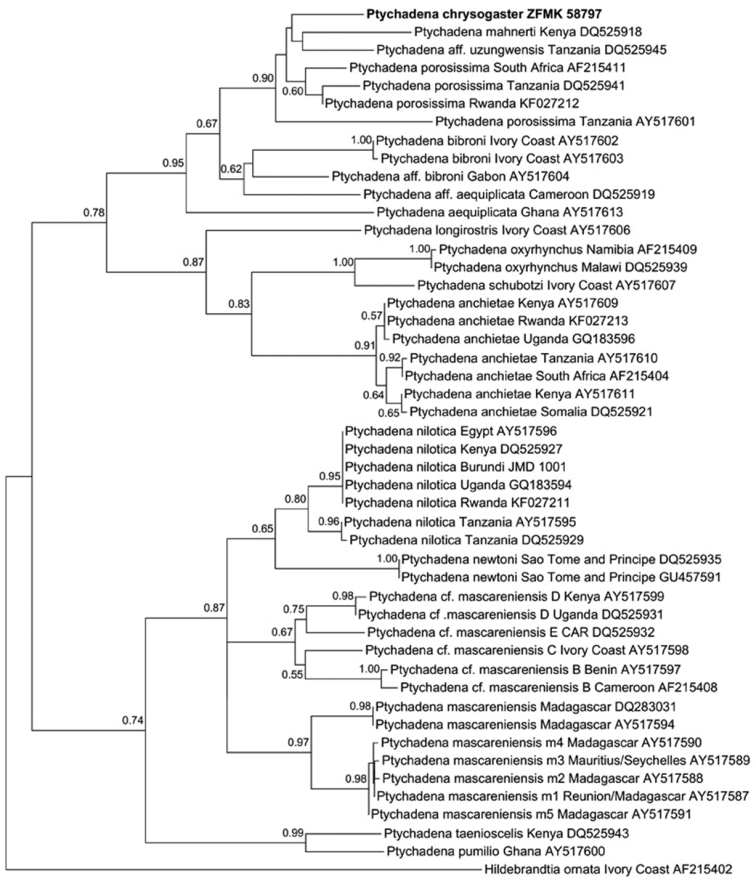
Maximum likelihood phylogram of species in the genus *Ptychadena* and *Hildebrandtia ornata* as outgroup, based on comparison of 548 base pairs of the mitochondrial 16S rRNA gene. Included are specimens from Rwanda and samples taken from GenBank (see Dehling and Sinsch in press for a complete list of sequences and accession numbers). Numbers above nodes are bootstrap support values (only values >0.50 are shown).

Comparison of the mitochondrial 16S rRNA gene sequences corroborated the status of *Ptychadena chrysogaster* as a distinct species. The partial sequence of this species differed from all available comparative sequences by an uncorrected p distance of at least 4.2 %. The p distance to sequences from Rwandan specimens of *Ptychadena anchietae* and *Ptychadena nilotica* was 13.1–13.3 % and 13.6 %, respecitively. The lowest values were observed in comparison with specimens assigned to “*Ptychadena* aff. *uzungwensis*” (4.2 %), “*Ptychadena porosissima*” from South Africa and Rwanda (4.7–4.9 %), “*Ptychadena* aff. *porosissima*” from Tanzania (6.0–6.9 %), “*Ptychadena mahnerti*” (6.2 %), and *“P.* aff. *bibroni*” from Gabon (6.9 %). The consensus tree yielded by Maximum-Likelihood analysis indicated that *Ptychadena chrysogaster* is most closely related to the aforementioned species (Figure 6). The clade consisting of these species is well supported by bootstraping (value 0.90; Hillis and Bull 1993), whereas the relationships within the clade are not resolved (bootstrap values <50 %).

## Discussion

Among the examined material we identified five distinct species of *Ptychadena*: *Ptychadena anchietae*, *Ptychadena chrysogaster*, *Ptychadena nilotica*, *Ptychadena porosissima*, and *Ptychadena uzungwensis*. The five species are distinguishable from each other unambiguously using quantitative morphometric as well as qualitative morphological characters. The comparison of the partial 16S rRNA sequence of a specimen of *Ptychadena chrysogaster* with sequences from congeners corroborated its distinct specific status. Sequences from specimens of *Ptychadena anchietae*, *Ptychadena nilotica*, and *Ptychadena porosissima* from Rwanda differ from each other considerably by an uncorrected p distance of more than 10 % (Sinsch et al. 2012, Dehling and Sinsch in press). Unfortunately, no homologous sequence of *Ptychadena uzungwensis* from Rwanda and only a sequence of a specimen with doubtful identity from Tanzania (*Ptychadena* aff. *uzungwensis*, GenBank# DQ525945) were available for comparison.

We did not find any *Ptychadena* individual collected in Rwanda which was assignable to *Ptychadena oxyrhynchus* in the collections of the RMCA and the ZFMK. Three specimens from Kisenyi (= Gisenyi, nowadays Rubavu; RMCA 51565–67), Rwanda, had been deposited under the name *Ptychadena oxyrhynchus* but were re-identified as males of *Ptychadena anchietae*. Nieden (1913) reported on several specimens of *Ptychadena oxyrhynchus* (as *Rana oxyrhyncha*) which Schubotz had collected in what today is northwestern Tanzania, at Kifumbiro and in the Mpororo area, close to the present border with Rwanda. We have not examined Schubotz’ material but if his specimens are indeed *Ptychadena oxyrhynchus* it is possible that the species can be found in Rwanda as well, given its vast distribution in eastern Africa and the fact that the herpetofauna of the northeastern part of Rwanda has been poorly sampled so far.

There is no specimen of *Ptychadena grandisonae* among the material Laurent collected in Rwanda. Laurent (1954) described the species based on type specimens from Muita in Angola, from Kanzenze and Kansenia in Katanga (DRC), and from Bitare in “Urundi” [= Burundi]. The latter is a town in central Burundi, about 20 km north of Gitega at 3°15'S, 29°54'E. Several authors, however, have stated that *Ptychadena grandisonae* occurs in Rwanda (Poynton and Broadley 1985, Channing 2001, Poynton and Channing 2004), and Poynton and Channing (2004) even stated that there is no record from Burundi. This misinformation was very likely caused by Laurent himself in a paper which was cited by all above mentioned authors, an account on the “Reptiles et Amphibiens de l’Angola” (Laurent 1964). Therein, Laurent (1964: 139) cited one of the type localities of *Ptychadena grandisonae* wrongly as “Bitare (Ruanda)”. On the same page, the locality is correctly given as “Bitare […] (Urundi)” in the account on *Ptychadena uzungwensis*. Thus, the often cited record of *Ptychadena grandisonae* from Rwanda in fact refers to specimens from Burundi (RMCA 109036–37; Appendix 1). So far, there is no evidence for the occurrence of *Ptychadena grandisonae* in Rwanda.

The available evidence indicates that only five species of *Ptychadena* occur in Rwanda. At present, three of these species (*Ptychadena anchietae*, *Ptychadena nilotica*, and *Ptychadena porosissima*) are widespread and can be found abundantly in both wetlands of the eastern lowland between 1300 and 2000 m elevation which drains into the Nile River and the western lowland on the shore of Lake Kivu which drains into the Congo River. The species inhabit higher elevations of up to 2300 m in deforested, cultivated areas, but are absent from dense forest habitats at similar elevations which at present only remain in the Volcano and Nyungwe National Parks and in the Gishwati Forest. *Ptychadena uzungwensis* is known from Rwanda from only few specimens, five males from “Kumunini” [= Munini, South Province, 2°42'S, 29°32'E] and a female from “Astrida” [= Butare/Huye, South Province, 2°36'S, 29°44'E], collected in 1952 and 1951, respectively, and has not been found since. Assuming the species is still extant in Rwanda, its distribution is apparently restricted to the south of the country.

There are large series of *Ptychadena chrysogaster* from various localities in Rwanda in the collection of the RMCA (Appendix 1), collected by Laurent in 1951–1952, indicating that the species was abundant at that time. We repeatedly conducted surveys at several of these localities including the type locality at Lac Karago (1°37'S, 29°30'E) but did not encounter individuals of *Ptychadena chrysogaster*. Our survey periods (February to April, September to October) were at similar times of the year to those of Laurent (Janurary, February, and October). Species of *Ptychadena* are among the most conspicuous frogs in areas they inhabit, usually occurring in high numbers and easy to detect. Although the absence of a species from a certain area cannot be proven ultimately, our observations indicate that *Ptychadena chrysogaster* has disappeared from these areas or at least is much less common than it used to be. The human population in Rwanda has grown from little more than 2 million people in 1950 to approximately 11 million in 2011 (United Nations, Population Division 2011). Nowadays almost every cultivatable area except the three national parks and few small forest patches has been altered to farmland (pers. observation). Gishwati Forest has been reduced to a small patch of a few square kilometres, but until the mid-1990s it had covered a large area in northwestern Rwanda and its extensions reached the shores of Lac Karago. The former presence of forest habitat at the lake is still indicated by the occurrence of two forest-dwelling frog species, *Hyperolius castaneus* and *Leptopelis kivuensis*,which call from bushes and groups of small trees at the shore of the river (own unpublished data; see also Sinsch et al. 2011). Judging from its collection sites, *Ptychadena chrysogaster* appears to occur primarily in wetlands within or at the edge of forest. Instead of *Ptychadena chrysogaster*, we found *Ptychadena nilotica* at Lac Karago and *Ptychadena nilotica* and *Ptychadena anchietae* in Huye (formerly Astrida and Butare) and in the vicinity of Muzanze (formerly Ruhengeri) during our recent surveys, two species that Laurent had not collected in Rwanda. Both species are known to be able to cope with habitat alteration and are often found in disturbed habitats and in human settlements (Spawls et al. 2006, pers. observation). It is possible that habitat alteration promoted population decline in *Ptychadena chrysogaster* and its replacement by otherspecies. The distribution of *Ptychadena chrysogaster* in Rwanda is currently under study. If our preliminary observations are affirmed, the Red List classification of *Ptychadena chrysogaster* would have to be changed to a “threatened” category and it would call for conservation measures.

Our recent efforts to untangle the diversity of *Ptychadena* in Rwanda are a first step to clarify the complicated taxonomy of the genus in sub-Saharan Africa. The results of our studies show that species of *Ptychadena* can be easily distinguished, if standardized diagnostic schemes are applied, which has also been demonstrated by previous studies (e.g. Perret 1979, Bwong et al. 2009). Integrative approaches combining data from morphology, bioacoustics, and molecular genetics will be the best way to address the existing taxonomic problems. Doubtful delimitations of *Ptychadena* species were often caused by assigning specimens to the wrong species based on non-diagnostic characters. Thereby, states of possible diagnostic characters were mixed up in subsequent accounts on these species, rendering them difficult to distinguish from each other. In the case of *Ptychadena chrysogaster*, two of its paratypes were in fact *Ptychadena porosissima*, a severe confusion by Laurent (1954). The latter species, however, was described by Laurent in the same paper as yet another new species, *Ptychadena loveridgei*. When even the describer cannot reliably distinguish the species, subsequent workers must fail. When reviewing the taxonomic status of certain species, it is mandatory to critically question decisions made by earlier authors by carefully re-evaluating proposed diagnostic characters and re-examining not only the holotypes, but also the material on which accounts discussing the variation within species and keys to species were based.

## Appendix 1

### Material examined

*Ptychadena anchietae*: Butare [= Huye], Rwanda (ZFMK 94575–89; twelve males, three females); “Kisenyi (Kivu)” [= Gisenyi/Rubavu, Rwanda] (RMCA 51565–67; three males).

*Ptychadena chrysogaster*: „Lac Karago, alt. 2250 m, terr. De Kisenyi“, Rwanda (RMCA 109096, one female, holotype; RMCA 109097–109113, sixteen males, paratypes); “Lulenga, Kivu”, DRC (RMCA 3452–69, eleven males, one female, paratypes; 2518–2521, four males, paratypes; 2759–68, one female, nine males, paratypes; 1748–55, seven males, one female, paratypes); “Gatsibu”, Rwanda (RMCA 36844, one male, paratype; 36866, one male, paratype); “Ruhengeri, riv. Moklungwa, alt. 1800–1825 m.), Ruanda” (RMCA 42011–13, three females, paratypes); “Lac Gando, alt. 2400 m, Ruanda” (RMCA 42025–28, four males, paratypes); “Region de Mulera, alt. 1800–2000 m”, Rwanda (RMCA 41987–88, one male, one female, paratypes); “Kasenze (versant S. Karisimbi)”, Rwanda (RMCA 42022–23, two males, paratypes); “entre Managna et Tshengelero, alt. 1750–2000 m”, DRC (RMCA 41965–41970, four males, one female, paratypes); “Kundhuru-Tshuve (col. Gahinga-Sabinyo), alt. 2600 m”, Rwanda (RMCA 41982–83, one male, one female, paratypes; RMCA 41984–86, three males, paratypes); “Ruhengeri, sources Kirii, alt. 1800–1825 m”, Rwanda (RMCA 41991, female, 41992–93, two males, paratypes); “Riv. Rodahira, afflt. de la riv. Fuku, s/afflt. de la riv. Rutshuru, près de Rutshuru, alt. 1250 m”, DRC (RMCA 116959, one male); “Nyabitsindi, entre le Visoke et le Musule, alt. 2400 m”, Rwanda (RMCA 42016–17, one male, one female, paratypes); “Kibga, riv. Suza, versant Sud Visoke, alt. 2400 m”, Rwanda (RMCA 42018, one male, paratype); “Dubi versant S. Visoke, Ruanda” (RMCA 42019–21, one female, two males, paratypes); “Shamuheru, Nyamuragira, alt. 1843 m”, DRC (RMCA 42031–32, one male, one female, paratypes); “Munagana, marais de Maziba, alt 2000 m”, Uganda (RMCA 41979–80, one male, one juvenile, paratypes); “Ilega (versant S. Karisimbi), Rubinda, alt. 2400 m”, Rwanda (RMCA 42024, subadult, paratype); “Kagogo, Lac Bulera, terr. de Ruhengeri, alt. 1870 m (Ruanda)” (RMCA 109121, one female; 109122, one male, paratypes); “Remera, Lac Luhondo, alt. 1770 m, terr. de Ruhengeri (Ruanda)” (RMCA 109114–120, three males, four females, paratypes); “Mutabonika, près de Ngabitsindi entre le Visoke et le Musule, alt. 2400 m” (RMCA 42014, one female; 42015, one male; paratypes); “Bitare, alt. 1650 m, Terr. de Kitega, Urundi” [= Burundi] (RMCA 109161–62, two males, paratypes); “Vyuya, Terr. de Bururi, alt. 1900 m (Urundi)” [=Burundi] (RMCA 109163–66, two males, paratypes); “Astrida, alt. 1750 m. (Ruanda)” [=Butare/Huye, Rwanda] (RMCA 109139–141, two females, one male, paratypes); “Tare, Busanza, région d’Astrida, alt. 1700 m (Ruanda)” (RMCA 109142, one female; 109143–147, four males, one female, paratypes); Mugatemba, Pref. Gikongoro, Rwanda (ZFMK 58747, one female); Nyungwe-Wald, Mukina (Kitabi), Rwanda (ZFMK 58797, one male); Cyamudongo, Rwanda (ZFMK 58847–850); Nyakalengijo, Mt Ruwenzori, Uganda (ZFMK 63239–241).

*Ptychadena grandisonae*: “Muita-Luembe, E (Angola)” (RMCA 60530, one female, holotype); ““Bitare, alt. 1650 m, Terr. de Kitega, Urundi” [= Burundi] (RMCA 109036–37, one male, one female, paratypes).

*Ptychadena guibei*: “Muita-Luembe, E (Angola)“ (RMCA 60535, female, holotype).

*Ptychadena nilotica*: Butare, Rwanda (ZFMK 94590–612, thirteen males, ten females; JMD 807, one male; EL 15-17, 25-26, 37, one male, five females); Mashyuza, Nyakabuye, Rwanda (ZFMK 58839–846); Route Kigali – Byumba, Rwanda (ZFMK 58851–852); Cyangugu, Rwanda (JMD 961, one male); Lac Karago, Rwanda (JMD 1028-1029); Bugarama, Rwanda (JMD 1074, one male); Bururi, Burundi (JMD 1001-1002, two males); Ruzizi National Park, Burundi (JMD 1015-1016, one male, one female). *[JMD& EL specs currently being assigned ZFMK nos.]*

*Ptychadena porosissima*: Butare [= Huye], Rwanda (ZFMK 94613–24, eleven males, one female); “Tare, reg. Astrida”, Rwanda (RMCA 109038, one female, holotype of *Ptychadena loveridgei* Laurent, 1954); “Astrida, alt. 1750 m” [= Huye], Rwanda (RMCA 109085–095, seven males, four females, paratypes of *Ptychadena loveridgei*); “Tare, Busanza”, Rwanda (RMCA 109039, one female, paratype of *Ptychadena loveridgei*); “Karambi, Terr. De Nyanza”, Rwanda (RMCA 109051–056, two females, paratypes of *Ptychadena loveridgei*); “Ruhengeri, riv. Moklungwa, alt. 1800–1825 m., Ruanda” (RMCA 42003–10, eight males, paratypes of *Ptychadena loveridgei*); “Ruhengeri, sources Kirii, alt. 1800–1825 m”, Rwanda (RMCA 41994, paratype of *Ptychadena chrysogaster*); “Reg. du Rwankeri, alt. 2200 m, Ruanda” (RMCA 41989, one male, paratype of *Ptychadena chrysogaster*).

*Ptychadena taenioscelis*: “Lukulu près Kiambi”, DRC (RMCA 13122, one subadult female, holotype).

*Ptychadena uzungwensis*: „Dabaga, Utschungwe Mts.“, Tanzania (RMCA 58843, one male, paratype); “Kumunini, Buyenza, alt. 2000 m, terr. d’ Astrida (Ruanda)” [= Munini, Rwanda] (RMCA 108993–97, five males); “Astrida, marais de la Mukura, alt. 1700 m (Ruanda)” [= Huye, Rwanda] (RMCA 108992, one female); “Bitare, alt. 1650 m, Terr. de Kitega, Urundi” (RMCA 108999–109016).

## Appendix 2

### Morphological features of *Ptychadena* spp. from Rwanda

The external morphology of each species is described in the following account. Descriptions are primarily based on Rwandan material. In case of *Ptychadena chrysogaster* we also included data from type specimens from Burundi, Uganda, and the Democratic Republic of the Congo. The description of *Ptychadena uzungwensis* is based on all available material from Rwanda and a male paratype from the type locality (see Appendix 1).

### Species accounts

***Ptychadena anchietae***

Body moderately sturdy, widest at temporal region, slightly tapering to groin; head large (HL/SUL 0.36–0.43 in males, 0.37–0.41 in females; HW/SUL 0.31–0.39 in males, 0.33–0.36 in females), longer than wide (HL/HW 1.01–1.26 in males, 1.11–1.13 in females); snout long (SL/HL 0.43–0.53 in males, 0.49–0.53 in females), pointed in dorsal view, rounded in profile, considerably projecting beyond lower jaw, longer than wide (SL/EE 1.08–1.24 in males, 1.30–1.33 in females); canthus rostralis distinct between eye and nostril, straight-lined; loreal region oblique, strongly concave; nostrils rounded, directed dorsolaterally; situated half-way between tip of snout and eye or closer to tip of snout than to eye (EN/NS 0.98–1.33 in males, 1.14–1.27 in females), separated from each other by distance subequal to distance between eye and nostril (NN/EN 0.87–1.05 in males, 0.85–0.96 in females); eyes directed anterolaterally, moderately protruding, relatively small (ED/HL 0.24–0.29 in males, 0.26–0.29 in females), its diameter much shorter than snout (ED/SL 0.50–0.63 in males, 0.53–0.58 in females); interorbital distance more or less equalling upper eyelid width (IO/EW 0.83–1.10 in males, 0.89–1.06 in females) and smaller than internarial distance (IO/NN 0.59–0.83 in males, 0.63–0.67 in females); tympanum and its annulus distinctly visible, separated from eye by about one-fifth to one-third of its diameter (ET/TD 0.21–0.36 in males, 0.19–0.25 in females); tympanum diameter 0.62–0.92 (in males) and 0.74–0.83 (in females) of eye diameter; upper jaw with dentition; choanae small, rounded, located far anterolaterally at margins of roof of mouth; vomer teeth in two short rows, separated from each other by distance about three times length of individual row; tongue long and narrow, bilobed for about one-sixth of its length, free distally for one-fourths its length; median lingual process absent; vocal sac in males paired, lateral; external vocal sac aperture as a longitudinal, posterolaterally orientated slit, inferior, terminating at level of ventral edge of insertion of arms; internal vocal sac apertures rounded, situated close to corner of mouth.

Dorsal surfaces of head, trunk and limbs finely shagreened with many scattered small tubercles; dorsum with five or six longitudinal dermal ridges on each side; median ridge extending from interorbital region almost to vent, postpalpebral and external ridges from level just behind posterior edge of upper eyelid to insertion of leg; laterodorsal ridge extending from level about one snout length posterior to tympanum to groin; dorsal ridges interrupted in few specimens; sacral ridge extending from about one head length anterior to vent either medially to median ridges or between median and postpalpebral ridges to vent, in few specimens absent; external ridge forming anterior part of supratympanic fold; posterior part of supratympanic fold less distinct, branching off from external dorsal ridge posterior to tympanum in wide angle and extending posterolaterally to insertion of arm; infratympanic fold thick and conspicuous, almost straight-lined, extending from ventral edge of eye to level of arm insertion, meeting with supratympanic fold; ventral side of limbs and body smooth except slightly areolate postaxial side of thigh; distinct transverse fold between arms on ventral side; supratympanic fold moderately distinct, angled, extending from posterior corner of eye to point dorsal from arm insertion; infratympanic fold thick and conspicuous, almost straight-lined, extending from ventral edge of eye to level of arm insertion, meeting with supratympanic fold, continued after a small gap in form of large oval tubercle dorsally of posterior end of arm insertion.

Forelimbs moderately sturdy; hand relatively small (HND/SUL 0.23–0.27 in males, 0.25–0.26 in females); tips of fingers rounded, not enlarged into disks but slightly swollen volarly; transverse dorsal skin ridge separating ultimate from other phalanges on each finger; relative length of fingers: I ≤ II < IV < III; subarticular tubercles rounded, well developed, numbering one on Fingers I and II, two on Fingers III and IV, proximal tubercles on Fingers III and IV larger and more prominent than distal ones; finger webbing absent; thenar tubercle distinct, large, flat, oval, slightly more than half as long as metacarpal of Finger I; inner palmar tubercle on proximal half of metacarpal region of Fingers II and III, rounded, flat; outer palmar tubercle on proximal half of metacarpal region of Finger IV, oval, slightly more prominent than inner palmar tubercle; one supernumerary metacarpal tubercle between palmar or thenar tubercles and proximal subarticular tubercles on each finger; callous longitudinal ridges between subarticular tubercles on Fingers III and IV and between subarticular tubercles and finger tips on all fingers; nuptial pads in males covering almost entire dorsal surfaces of Fingers I and II, and proximal portion of dorsal side of Finger III.

Hindlimbs sturdy, very long (LEG/SUL 1.87–2.11 in males, 1.96–2.13 in females); knee reaching slightly beyond insertion of forelimbs and tibio-tarsal articulation reaching almost a head length beyond tip of snout when legs are adpressed forwardly to body; tibiofibula very long (TFL/SUL 0.61–0.68 in males, 0.65–0.72 in females), longer than thigh (TFL/THL 1.09–1.19 in males, 1.13–1.16 in females); heels overlapping each other considerably when knees flexed and thighs held perpendicularly to median plane; two low longitudinal ridges on plantar side of tarsus between heel and metatarsal tubercles; foot shorter than or equal in length to tibiofibula (FOT/TFL 0.85–1.00 in males, 0.91–0.93 in females); relative length of toes: I < II < III < V < IV; toe tips rounded, not enlarged into disks but slightly swollen plantarly; transverse dorsal skin ridge separating ultimate from other phalanges on each toe; subarticular tubercles numbering one on Toes I and II, two on Toes III and V, and three on Toe IV; low callous ridges between subarticular tubercles, and between subarticular tubercles and toe tips; pedal webbing formula **I** 0.5-2**II** 0.5-2**III**(0.5–1)-2**IV** 2-0.5**V**; inner metatarsal tubercle moderately large, half as long as metatarsus of Toe I, oval, prominent; outer metatarsal tubercle rounded, flat, faintly visible, callous tissue weakly developed.

***Ptychadena chrysogaster* Laurent, 1954**

Body moderately sturdy, widest at temporal region, slightly tapering to groin; head large (HL/SUL 0.34–0.38 in males, 0.33–0.37 in females; HW/SUL 0.29–0.34 in males, 0.28–0.34 in females), longer than wide (HL/HW 1.04–1.22 in males, 1.05–1.23 in females); snout long (SL/HL 0.45–0.50 in males, 0.45–0.51 in females), pointed in dorsal view, rounded in profile, considerably projecting beyond lower jaw, longer than wide (SL/EE 1.11–1.28 in males, 1.11–1.37 in females); canthus rostralis distinct between eye and nostril, straight-lined; loreal region oblique, strongly concave; nostrils rounded, directed dorsolaterally; situated more or less half-way between tip of snout and eye (EN/NS 0.87–1.14 in males, 0.93–1.24 in females), separated from each other by distance subequal to or larger than distance between eye and nostril (NN/EN 1.02–1.22 in males, 0.94–1.24 in females); eyes directed anterolaterally, moderately protruding, relatively small (ED/HL 0.27–0.32 in males, 0.26–0.30 in females), its diameter much shorter than snout (ED/SL 0.57–0.66 in males, 0.51–0.66 in females); interorbital distance larger than upper eyelid width (IO/EW 1.12–1.48 in males, 1.09–1.44 in females) and smaller than internarial distance (IO/NN 0.75–0.89 in males, 0.78–0.91 in females); tympanum and its annulus distinctly visible, separated from eye by about one-fourth to two-fifths of its diameter (ET/TD 0.24–0.38 in males, 0.26–0.40 in females); tympanum diameter slightly smaller to subequal to eye diameter (TD/ED 0.76–0.98 in males, 0.85–1.01 in females); upper jaw with dentition; choanae small, rounded, located far anterolaterally at margins of roof of mouth; vomer teeth in two short rows, separated from each other by distance about three times length of individual row; tongue long and narrow, bilobed for about one-fifth of its length, free distally for one-fourth its length; median lingual process absent; vocal sac in males paired, lateral; external vocal sac aperture as a longitudinal, posterolaterally orientated slit, inferior, terminating at level of ventral edge of ventral insertion of arms; internal vocal sac apertures rounded, situated close to corner of mouth.

Dorsal surfaces of head, trunk and limbs finely shagreened; dorsum with five or six longitudinal dermal ridges on each side, median one extending from interorbital region almost to vent, postpalpebral and external ones from level just behind posterior edge of upper eyelid to insertion of leg; laterodorsal ridge extending from level about one snout length posterior to tympanum to groin; sacral ridge extending from about one head length anterior to vent either medially to median ridges or between median and postpalpebral ridges to vent; in few specimens additional sacromedial ridge extending between sacral ridge and median ridge to vent for about half length of sacral ridge; external ridge forming anterior part of supratympanic fold; posterior part of supratympanic fold less distinct, branching off from external dorsal ridge posterior to tympanum in wide angle and extending posterolaterally to insertion of arm; infratympanic fold thick and conspicuous, almost straight-lined, extending from ventral edge of eye to level of arm insertion, meeting with supratympanic fold; ventral side of limbs and body smooth except areolate proximal postaxial-ventral part of thigh; distinct transverse fold between arms on ventral side; ventral side of trunk and head densely covered with more or less evenly scattered tiny, pointed tubercles in males.

Forelimbs moderately sturdy; hand relatively small (HND/SUL 0.22–0.26 in males, 0.21–0.25 in females); tips of fingers rounded, not enlarged into disks but slightly swollen volarly; transverse dorsal skin ridge separating ultimate from other phalanges on each finger; relative length of fingers: I = II < IV < III; subarticular tubercles rounded, well developed, numbering one on Fingers I and II, two on Fingers III and IV, proximal tubercles on Fingers III and IV larger and more prominent than distal ones; finger webbing absent; thenar tubercle prominent, elongated, large, two-thirds length of metacarpal of Finger I; inner palmar tubercle on proximal third of metacarpal region of Fingers II and III, oval, flat; outer palmar tubercle on proximal half of metacarpal region of Finger IV, elongated, slightly more prominent than inner palmar tubercle; one supernumerary metacarpal tubercle between palmar or thenar tubercles and proximal subarticular tubercles on all fingers; low callous longitudinal ridges between subarticular tubercles on Fingers III and IV and between subarticular tubercles and finger tips on all fingers; nuptial pads in males covering almost entire dorsal surfaces of Fingers I and II except distal phalanx, and preaxial half of dorsal side of metacarpal of Finger III.

Hindlimbs sturdy, very long (LEG/SUL 1.94–2.27 in males, 1.93–2.14 in females); knee reaching to insertion of forelimbs and tibio-tarsal articulation reaching slightly more than a snout length beyond tip of snout when legs adpressed forwardly to body; tibiofibula very long (TFL/SUL 0.61–0.73 in males, 0.62–0.70 in females), longer than thigh (TFL/THL 1.13–1.22 in males, 1.13–1.25 in females); heels overlapping each other considerably when knees flexed and thighs held perpendicularly to median body plane; two low longitudinalridges on plantar side of tarsus between heel and metatarsal tubercles; foot subequal in length to tibiofibula (FOT/TFL 0.97–1.07 in males, 0.94–1.03 in females); relative length of toes: I < II < III < V < IV; toe tips rounded, not enlarged into disks but slightly swollen plantarly; transverse dorsal skin ridge separating ultimate from other phalanges on each toe; subarticular tubercles numbering one on Toes I and II, two on Toes III and V, and three on Toe IV; low callous ridges between subarticular tubercles, and between subarticular tubercles and toe tips; pedal webbing formula **I** 2-2.5**II**(1.5–1.75)-3**III**(2–2^-^)(3.25–3^+^)**IV** 3-(1.5–2)**V**; inner metatarsal tubercle elongated, prominent, small, less than half as long as metatarsus of Toe I; outer metatarsal tubercle very faintly visible, rarely prominent, small and pointed.

***Ptychadena nilotica***

Body moderately sturdy, widest at temporal region, slightly tapering to groin; head large (HL/SUL 0.35–0.43 in males, 0.35–0.39 in females; HW/SUL 0.30–0.37 in males, 0.32–0.35 in females), longer than wide (HL/HW 1.10–1.27 in males, 1.06–1.15 in females); snout long (SL/HL 0.38–0.46 in males, 0.40–0.45 in females), pointed in dorsal view, rounded in profile, considerably projecting beyond lower jaw, longer than wide (SL/EE 1.20–1.40 in males, 1.15–1.36 in females); canthus rostralis distinct between eye and nostril, straight-lined; loreal region oblique, moderately concave; nostrils rounded, directed dorsolaterally; situated half-way between tip of snout and eye or closer to tip of snout than to eye (EN/NS 0.99–1.18 in males, 1.05–1.34 in females), separated from each other by distance slightly less than or subequal to distance between eye and nostril (NN/EN 0.82–0.99 in males, 0.87–0.97 in females); eyes directed anterolaterally, moderately protruding, relatively small (ED/HL 0.25–0.32 in males, 0.26–0.29 in females), its diameter shorter than snout (ED/SL 0.60–0.69 in males, 0.59–0.69 in females); interorbital distance smaller to equalling upper eyelid width (IO/EW 0.64–1.03 in males, 0.72–0.90 in females) and smaller than internarial distance (IO/NN 0.55–0.74 in males, 0.57–0.72 in females); tympanum and its annulus distinctly visible, separated from eye by about one-fourth to one-third of its diameter (ET/TD 0.28–0.36 in males, 0.26–0.37 in females); tympanum diameter 0.75–0.88 (in males) and 0.77–0.89 (in females) of eye diameter; upper jaw with dentition; choanae small, rounded, located far anterolaterally at margins of roof of mouth; vomer teeth in two short rows, separated from each other by distance about three times length of individual row; tongue long and narrow, bilobed for about one-sixth of its length, free distally for one-third its length; median lingual process absent; vocal sac in males paired, lateral; external vocal sac aperture as a longitudinal, posteriorly orientated slit, situated dorsally of level of insertion of arms, parallel to mandibel and infratympanic fold, covered by narrow dermal flap on ventral edge of slit; internal vocal sac apertures rounded, situated close to corner of mouth.

Dorsal surfaces of head, trunk and limbs shagreened; dorsum with four or five longitudinal dermal ridges on each side, median one extending from interorbital region almost to vent, postpalpebral and external ones from level just behind posterior edge of upper eyelid to insertion of leg; laterodorsal ridge extending from level about one snout length posterior to tympanum to groin; in few specimens additional very short ridge present between median and postpalpebral ridge from about one snout length anterior to vent extending almost to vent; external ridge forming anterior part of supratympanic fold; posterior part of supratympanic fold less distinct, branching off from external dorsal ridge posterior to tympanum in wide angle and extending posterolaterally to insertion of arm; infratympanic fold thick and conspicuous, almost straight-lined, extending from ventral edge of eye to level of arm insertion, meeting with supratympanic fold; ventral side of limbs and body smooth except areolate postaxial side of thigh; distinct transverse fold between arms on ventral side.

Forelimbs moderately sturdy; hand relatively small (HND/SUL 0.21–0.27 in males, 0.22–0.26 in females); tips of fingers rounded, not enlarged into disks but slightly swollen volarly; transverse dorsal skin ridge separating ultimate from other phalanges on each finger; relative length of fingers: I ≤ II < IV < III; subarticular tubercles rounded, well developed, numbering one on Fingers I and II, two on Fingers III and IV; proximal tubercles on Fingers III and IV larger and more prominent than distal ones; finger webbing absent; thenar tubercle prominent, elongated and narrow, comparatively small, less than half as long as metacarpal of Finger I; inner palmar tubercle small, flat, less conspicuous, on proximal one-third to one-fourth of metacarpal region of Fingers II and III, rounded, flat; outer palmar tubercle on proximal half of metacarpal region of Finger IV, prominent, elongate and narrow; single supernumerary metacarpal tubercle between outer palmar tubercle and proximal subarticular tubercle of Finger IV, often indistinct; callous longitudinal ridges between subarticular tubercles on Fingers III and IV and between subarticular tubercles and finger tips on all fingers; nuptial pads in males covering almost entire dorsal surfaces of Fingers I and II and preaxial half of dorsal surface of Finger III.

Hindlimbs sturdy, long (LEG/SUL 1.71–1.97 in males, 1.76–1.96 in females); knee reaching just behind insertion of forelimbs and tibio-tarsal articulation reaching tip of snout when legs are adpressed forwardly to body; tibiofibula moderately long (TFL/SUL 0.50–0.60 in males, 0.54–0.60 in females), longer than thigh (TFL/THL 1.04–1.12 in males, 1.09–1.12 in females); heels overlapping each other when knees flexed and thighs held perpendicularly to median plane; two low longitudinalridges on plantar side of tarsus between heel and metatarsal tubercles; foot long, longer than tibiofibula (FOT/TFL 1.07–1.14 in males, 1.03–1.10 in females); relative length of toes: I < II < III < V < IV; toe tips rounded, not enlarged into disks but slightly swollen plantarly; transverse dorsal skin ridge separating ultimate from other phalanges on each toe; subarticular tubercles numbering one on Toes I and II, two on Toes III and V, and three on Toe IV; low callous ridges between subarticular tubercles, and between subarticular tubercles and toe tips; pedal webbing formula **I**(1.5–1.75)-(2–2.25)**II** 1.5-(2.75–3)**III**(1.75–2)-3**IV** 2.75-(1–1.5)**V**; inner metatarsal tubercle elongated, prominent, small, less than half as long as metatarsus of Toe I; outer metatarsal tubercle rounded, flat, distinctly present, rarely faintly visible.

***Ptychadena porosissima***

Body moderately sturdy, widest at temporal region, slightly tapering to groin; head large (HL/SUL 0.35–0.41 in males, 0.35–0.40 in females; HW/SUL 0.31–0.36 in males, 0.30–0.34 in females), longer than wide (HL/HW 1.02–1.19 in males, 1.03–1.21 in females); snout moderately long (SL/HL 0.38–0.49 in males, 0.43–0.51 in females), pointed in dorsal view, rounded in profile, considerably projecting beyond lower jaw, longer than wide (SL/EE 1.21–1.27 in males, 1.11–1.61 in females); canthus rostralis distinct between eye and nostril, straight-lined; loreal region oblique, strongly concave; nostrils rounded, directed dorsolaterally; situated more or less half-way between tip of snout and eye (EN/NS 0.89–1.15 in males, 0.87–1.29 in females), separated from each other by distance subequal to or larger than distance between eye and nostril (NN/EN 0.96–1.15 in males, 0.96–1.11 in females); eye directed anterolaterally, moderately protruding, relatively small (ED/HL 0.24–0.30 in males, 0.25–0.29 in females), its diameter much shorter than snout (ED/SL 0.54–0.67 in males, 0.51–0.63 in females); interorbital distance subequal to upper eyelid width (IO/EW 0.80–1.09 in males, 0.81–1.10 in females) and smaller than internarial distance (IO/NN 0.65–0.74 in males, 0.60–0.82 in females); tympanum and its annulus distinctly visible, separated from eye by about one-fourth to two-fifths of its diameter (ET/TD 0.27–0.39 in males, 0.29–0.39 in females); tympanum diameter smaller than eye diameter (TD/ED 0.68–0.81 in males, 0.70–0.84 in females); upper jaw with dentition; choanae small, rounded, located far anterolaterally at margins of roof of mouth; vomer teeth in two short rows, separated from each other by distance about three times length of individual row; tongue long and narrow, bilobed for about one-fifth of its length, free distally for one-third its length; median lingual process absent; vocal sac in males paired, lateral; external vocal sac aperture as a longitudinal, posterolaterally orientated slit, inferior, terminating at level of ventral edge of insertion of arms; internal vocal sac apertures rounded, situated close to corner of mouth.

Dorsal surfaces of head, trunk and limbs finely shagreened; dorsum with four or five longitudinal dermal ridges on each side; median ridge extending from interorbital region almost to vent, postpalpebral and external ridges from posterior edge of upper eyelid to insertion of leg; laterodorsal ridge extending from level of insertion of arm to groin; sacral ridge extending from about one head length anterior to vent to vent, in some specimens absent; external ridge forming anterior part of supratympanic fold; posterior part of supratympanic fold less distinct, branching off from external dorsal ridge posterior to tympanum in wide angle and extending posterolaterally to insertion of arm; infratympanic fold thick and conspicuous, almost straight-lined, extending from ventral edge of eye to level of arm insertion, meeting with supratympanic fold; ventral side of limbs and body smooth except areolate postaxial side of thigh; distinct transverse fold between arms on ventral side; ventral side of trunk and head densely covered with more or less evenly scattered small, pointed tubercles in males.

Forelimbs moderately sturdy; hand relatively small (HND/SUL 0.22–0.26 in males, 0.22–0.24 in females); tips of fingers rounded, not enlarged into disks but slightly swollen volarly; transverse dorsal skin ridge separating ultimate from other phalanges on each finger; relative length of fingers: I ≤ II < IV < III; subarticular tubercles rounded, well developed, numbering one on Fingers I and II, two on Fingers III and IV, proximal tubercles on Fingers III and IV larger and more prominent than distal ones; finger webbing absent; thenar tubercle distinct, elongated, large, two-thirds length of metacarpal of Finger I; inner palmar tubercle on proximal third of metacarpal region of Fingers II and III, oval, flat; outer palmar tubercle on proximal half of metacarpal region of Finger IV, elongated, slightly more prominent than inner palmar tubercle; one supernumerary metacarpal tubercle between palmar or thenar tubercles and proximal subarticular tubercles on Fingers I, II, and IV, two on Finger III; callous longitudinal ridges between subarticular tubercles on Fingers III and IV and between subarticular tubercles and finger tips on all fingers; nuptial pads in males covering almost entire dorsal surfaces of Fingers I and II and preaxial half of dorsal side of Finger III.

Hindlimbs sturdy, very long (LEG/SUL 1.77–2.05 in males, 1.80–2.06 in females); knee reaching to insertion of forelimbs and tibio-tarsal articulation reaching slightly more than one snout length beyond tip of snout when legs adpressed forwardly to body; tibiofibula long (TFL/SUL 0.56–0.65 in males, 0.56–0.66 in females), longer than thigh (TFL/THL 1.08–1.18 in males, 1.05–1.26 in female); heels overlapping each other considerably when knees flexed and thighs held perpendicularly to median plane; two low longitudinal ridges on plantar side of tarsus between heel and metatarsal tubercles; foot slightly shorter than or equal in length to tibiofibula (FOT/TFL 0.94–1.01 in males, 0.92–1.01 in females); relative length of toes: I < II < III < V < IV; toe tips rounded, not enlarged into disks but slightly swollen plantarly; transverse dorsal skin ridge separating ultimate from other phalanges on each toe; subarticular tubercles numbering one on Toes I and II, two on Toes III and V, and three on Toe IV; low callous ridges between subarticular tubercles, and between subarticular tubercles and toe tips; pedal webbing formula **I**(1.75–2)-2.25**II** 1.5-3**III** 1.75-(3–3.25)**IV** 3-(1–1.5)**V**; inner metatarsal tubercle very prominent, shovel-like, large, more than half as long as metatarsus of Toe I; outer metatarsal tubercle faintly visible, rarely prominent and pointed.

***Ptychadena uzungwensis***

Body moderately sturdy, widest at temporal region, slightly tapering to groin; head large (HL/SUL 0.36–0.39 in males, 0.37 in females; HW/SUL 0.32–0.35 in males, 0.32 in female), longer than wide (HL/HW 1.06–1.23 in males, 1.16 in female); snout long (SL/HL 0.50–0.52 in males, 0.50 in female), pointed in dorsal view, rounded in profile, considerably projecting beyond lower jaw, much longer than wide (SL/EE 1.30–1.53 in males, 1.56 in female); canthus rostralis distinct between eye and nostril, straight-lined; loreal region oblique, strongly concave; nostrils rounded, directed dorsolaterally; situated more or less half-way between tip of snout and eye (EN/NS 0.91–1.03 in males, 0.97 in female), separated from each other by distance subequal to or shorter than distance between eye and nostril (NN/EN 0.82–0.94 in males, 0.89 in female); eyes directed anterolaterally, moderately protruding, relatively small (ED/HL 0.27–0.32 in males, 0.28 in female), its diameter much shorter than snout (ED/SL 0.55–0.63 in males, 0.56 in female); interorbital distance subequal to or larger than upper eyelid width (IO/EW 0.97–1.24 in males, 1.19 in female) and smaller or subequal to internarial distance (IO/NN 0.80–1.06 in males, 0.78 in female); tympanum and its annulus distinctly visible, separated from eye by about two-fifths to half of its diameter (ET/TD 0.36–0.47 in males, 0.41 in female); tympanum diameter smaller than eye diameter (TD/ED 0.70–0.83 in males, 0.77 in female); upper jaw with dentition; choanae small, rounded, located far anterolaterally at margins of roof of mouth; vomer teeth in two short rows, separated from each other by distance about two and half times length of individual row; tongue long and narrow, bilobed for about one-third of its length, free distally for one-third its length; median lingual process absent; vocal sac in males paired, lateral; external vocal sac aperture as a longitudinal, posterolaterally orientated slit, semi-inferior, terminating at level of centre of insertion of arms; internal vocal sac apertures rounded, situated close to corner of mouth.

Dorsal surfaces of head, trunk and limbs finely shagreened; dorsum with four longitudinal dermal ridges on each side; median ridge extending from dorsal side of snout between nostrils almost to vent; postpalpebral ridge extending from posterior portion of upper eyelid, external ridge from level of tympanum to insertion of leg; laterodorsal ridge extending from level of tympanum to groin; external ridge forming anterior part of supratympanic fold; posterior part of supratympanic fold less distinct, branching off from external dorsal ridge posterior to tympanum in wide angle and extending posterolaterally to insertion of arm; infratympanic fold thick and conspicuous, almost straight-lined, extending from ventral edge of eye to level of arm insertion, meeting with supratympanic fold; ventral side of limbs and body smooth except areolate postaxial side of thigh; distinct transverse fold between arms on ventral side; ventral side of trunk and head densely covered with more or less evenly scattered small, pointed, very small tubercles in males.

Forelimbs moderately sturdy; hand relatively small (HND/SUL 0.22–0.24 in males, 0.21 in female); tips of fingers rounded, not enlarged into disks but slightly swollen volarly; transverse dorsal skin ridge separating ultimate from other phalanges on each finger; relative length of fingers: II = IV ≤ I < III; subarticular tubercles rounded, well developed, numbering one on Fingers I and II, two on Fingers III and IV, proximal tubercles on Fingers III and IV larger and more prominent than distal ones; finger webbing absent; thenar tubercle distinct, elongated, large, half as long as metacarpal of Finger I; inner palmar tubercle on proximal third of metacarpal of Finger III, small, roundish, flat; outer palmar tubercle on proximal third of metacarpal of Finger IV, elongated, more prominent than inner palmar tubercle; one supernumerary metacarpal on Fingers I and IV, two on Finger II, and two to four on Finger III between palmar or thenar tubercles and proximal subarticular tubercles; callous longitudinal ridges between subarticular tubercles on Fingers III and IV and between subarticular tubercles and finger tips on all fingers; nuptial pads in males covering almost entire dorsal surfaces of metacarpal and proximal phalanx of Fingers I and II and preaxial halves of dorsal sides of metacarpal and [in Rwandan specimens but not in paratype and specimens from Kivu and Burundi] proximal portion of proximal phalanx of Finger III.

Hindlimbs sturdy, very long (LEG/SUL 1.84–2.04 in males, 1.87 in female); knee reaching to insertion of forelimbs and tibio-tarsal articulation reaching slightly more than one snout length beyond tip of snout when legs adpressed forwardly to body; tibiofibula long (TFL/SUL 0.59–0.66 in males, 0.62 in female), longer than thigh (TFL/THL 1.12–1.20 in males, 1.16 in female); heels overlapping each other considerably when knees flexed and thighs held perpendicularly to median body plane; two low longitudinal ridges on plantar side of tarsus between heel and metatarsal tubercles, postaxial one less distinct than preaxial one; foot shorter than or subequal in length to tibiofibula (FOT/TFL 0.94–1.03 in males, 1.07 in female); relative length of toes: I < II < III < V < IV; toe tips rounded, not enlarged into disks but slightly swollen plantarly; transverse dorsal skin ridge separating ultimate from other phalanges on each toe; subarticular tubercles numbering one on Toes I and II, two on Toes III and V, and three on Toe IV; low callous ridges between subarticular tubercles, and between subarticular tubercles and toe tips; pedal webbing formula **I** 2-(2.25–2.5)**II** 1.5-3^-^**III**(1.75–2^-^)-3**IV** 3-(1^+^–1.25)**V**; inner metatarsal tubercle prominent, elongated, large, half as long as metatarsus of Toe I; outer metatarsal tubercle faintly visible.

## References

[B1] Boulenger GA (1879) Synonymie de *Rana mascareniensis*. Bulletin de la Société Zoologique de France 4: 92-94.

[B2] Branch B (2005) A Photographic Guide to Snakes, other reptiles and amphibians of East Africa. Struik Publishers, Cape Town.

[B3] Bwong BA Chira R Schick S Veith M Lötters S (2009) Diversity of Ridged Frogs (Ptychadenidae: *Ptychadena*) in the easternmost remnant of the Guineo-Congolian rain forest: an analysis using morphology, bioacoustics and molecular genetics. Salamandra 45: 129-146.

[B4] Channing A (2001) Amphibians of central and southern Africa. Comstock Publishing Associates, Ithaca and London.

[B5] Channing A Howell KM (2006) Amphibians of East Africa. Chimaira, Frankfurt/M.

[B6] Dehling JM Sinsch U (in press) Diversity of Ridged Frogs (Anura: Ptychadenidae: *Ptychadena* spp.) in wetlands of the upper Nile in Rwanda: Morphological, bioacoustic, and molecular evidence. Zoologischer Anzeiger. doi: 10.1016/j.jcz.2013.08.005

[B7] Duméril AMC Bibron G (1841) Erpétologie générale ou histoire naturelle compléte de reptiles. Paris.

[B8] Fischer E Hinkel H (1992) Natur Ruandas / La Nature du Rwanda — Einführung in die Flora und Fauna Ruandas und ihre wichtigsten Biotopsysteme. Ministerium des Inneren und für Sport, Rheinland-Pfalz, Mainz.

[B9] Guibé J Lamotte M (1957) Révision systématique des *Ptychadena* (Batraciens, Anoures, Ranidés) d’Afrique occidentale. Bulletin de l’Institut Française d’Afrique Noire. Série A, Sciences Naturelles 19: 937-1003.

[B10] Hillis DM Bull JJ (1993) An empirical test of bootstrapping as a method for assessing confidence in phylogenetic analysis. Systematic Biology 42: 182-192.

[B11] Lamotte M (1967) Le problème des *Ptychadena* (Fam. Ranidae) du groupe mascareniensis dans l’ouest Africain. Bulletin du Muséum National d’Histoire Naturelle, 2e series 39: 647–656.

[B12] Laurent RF (1954) Etude de quelques espèces méconnues du genre *Ptychadena*. Annales du Musée Royal du Congo Belge Tervuren (Belgique) — Sciences Zoologiques 34: 1-34.

[B13] Laurent RF (1964) Museu do Dundo — Subsídios para o estudo da biologia na Lunda. Reptiles et Amphibiens de l’Angola (Troisème contribution). Publicações Culturais da Companhia de Diamantes de Angola 67: 11-165.

[B14] Loveridge A (1932) New reptiles and amphibians from Tanganyika Territory and Kenya Colony. Bulletin of the Museum of Comparative Zoology 72: 375-387.

[B15] Myers CW Duellman WE (1982) A new species of *Hyla* from Cerro Colorado, and other treefrog records and geographical notes from western Panama. American Museum Novitates 2752: 1-32.

[B16] Nieden F (1913) Amphibia. In: Schubotz H (Ed) Wissenschaftliche Ergebnisse der deutschen Zentral-Afrika-Expedition unter Führung Adolf Friedrichs, Herzogs zu Mecklenburg. Band IV, Zoologie II. Klinkhardt & Biermann, Leipzig, 165–195, plt. V.

[B17] Perret J-L (1979) Remarques et mise au point sur quelques espèces de *Ptychadena* (Amphibia, Ranidae). Bulletin de la Société neuchâteloise des Sciences naturelles 102: 5-21.

[B18] Poynton JC (1970) Guide to the *Ptychadena* (Amphibia: Ranidae) of the southern third of Africa. Annals of the Natal Museum 20: 365-375.

[B19] Poynton JC Broadley DG (1985) Amphibia Zambesiaca 2. Ranidae. Annals of the Natal Museum 27: 115-181.

[B20] Poynton J Channing A (2004) *Ptychadena grandisonae*. In: IUCN 2012. IUCN Red List of Threatened Species. Version 2012.2. www.iucnredlist.org

[B21] Poynton J Largen M Howell K Channing A Minter L Lötters S (2004) *Ptychadena anchietae*. In: IUCN 2012. IUCN Red List of Threatened Species. Version 2012.2. www.iucnredlist.org

[B22] Rödel M-O (2000) Herpetofauna of West Africa. Vol. 1. Amphibians of the West African savanna. Chimaira, Frankfurt/M.

[B23] Schmidt KP Inger RF (1959) Amphibians exclusive of the genera *Afrixalus* and *Hyperolius*. Exploration du Parc National de l’Upemba. Mission G.F. de Witte 56: 1-264.

[B24] Sinsch U Greenbaum E Kusamba C Lehr E (2011) Rapid assessment of montane anuran communities in the Albertine Rift: *Hyperolius castaneus* Ahl, 1931 as an umbrella species for conservation. African Zoology 46: 320-333. doi: 10.3377/004.046.0211

[B25] Sinsch U Lümkemann K Rosar K Schwarz C Dehling JM (2012) Acoustic niche partitioning in an anuran community inhabiting an Afromontane wetland (Butare, Rwanda). African Zoology 47: 60-73. doi: 10.3377/004.047.0122

[B26] Smith A (1849) Illustrations of the Zoology of South Africa; Consisting Chiefly of Figures and Descriptions of the Objects of Natural History Collected during an Expedition into the Interior of South Africa, in the Years 1834, 1835, and 1836... . Vol. III. Reptilia, Part 28. Smith, Elder & Co, London.

[B27] Spawls S Howell KM Drewes RC (2006) Pocket guide to the reptiles and amphibians of East Africa. A&C Publishers, London.

[B28] Tamura K Peterson D Peterson N Stecher G Nei M Kumar S (2011) MEGA5: Molecular Evolutionary Genetics Analysis Using Maximum Likelihood, Evolutionary Distance, and Maximum Parsimony Methods. Molecular Biology and Evolution 28: 2731-2739. doi: 10.1093/molbev/msr121 21546353 PMC3203626

[B29] United Nations, Department of Economic and Social Affairs, Population Division (2011) World Population Prospects: The 2010 Revision, CD-ROM Edition.

[B30] Vences M Thomas M van der Meijden A Chiari Y Vieites DR (2005) Comparative performance of the 16S rRNA gene in DNA barcoding of amphibians. Frontiers in Zoology 2: 1–12. doi: 10.1186/1742-9994-2-5 15771783 PMC555853

